# Endodontic Treatment of Maxillary Premolar with Three Root Canals Using Optical Microscope and NiTi Rotatory Files System

**DOI:** 10.1155/2013/710408

**Published:** 2013-12-03

**Authors:** João Bosco Formiga Relvas, Fredsom Marcio Acris de Carvalho, André Augusto Franco Marques, Emílio Carlos Sponchiado, Lucas da Fonseca Roberti Garcia

**Affiliations:** ^1^Department of Endodontics, School of Dentistry, Federal University of Amazonas, 69077-000 Manaus, AM, Brazil; ^2^Department of Endodontics, School of Dentistry, Paulista University, 69050-010 Manaus, AM, Brazil; ^3^Department of Endodontics, School of Dentistry, State University of Amazonas, 69050-030 Manaus, AM, Brazil; ^4^Department of Dental Materials and Prosthodontics, Ribeirão Preto School of Dentistry, University of São Paulo, 14040-900 Ribeirão Preto, SP, Brazil

## Abstract

The aim of the study was to report a clinical case of endodontic treatment of a maxillary first premolar with three root canals using an optical microscope and rotary instrumentation technique. The main complaint of the patient, a 16-year-old girl, was pain in tooth 14. After clinical and radiographic examination, irreversible pulpitis was diagnosed. An alteration in the middle third of the pulp chamber radiographically observed suggested the presence of three root canals. Pulp chamber access and initial catheterization using size number 10 K-files were performed. The optical microscope and radiographic examination were used to confirm the presence of three root canals. PathFiles #13, #16, and #19 were used to perform catheterization and ProTaper files S1 and S2 for cervical preparation. Apical preparation was performed using F1 file in the buccal canals and F2 in the palatal canal up to the working length. The root canals were filled with Endofill sealer by thermal compaction technique using McSpadden #50. The case has been receiving follow-up for 12 months and no painful symptomatology or periapical lesions have been found. The use of technological tools was able to assist the endodontic treatment of teeth with complex internal anatomy, such as three-canal premolars.

## 1. Introduction

The constant development of equipment and instrumentation techniques has made it possible to solve several clinical cases in the field of Endodontics [1]. However, it is still imperative that professionals have a thorough knowledge of the internal anatomy of the pulp chamber and the entire root canal system in order to increase efficiency and, consequently, the rate of clinical success of endodontic treatment [2, 3].

Among permanent teeth, the roots of maxillary first premolars often have two conical roots, that is one buccal and one palatal root, which may present root fusion with a distinct line between them [4]. The buccal root may be further divided into two, an incidence of 1% to 5%, which causes the tooth to have three canals, a palatal, a distobuccal, and a mesiobuccal canal [4, 5].

Despite the low incidence, several studies have demonstrated the existence of three-canal maxillary first premolars, which considerably makes endodontic treatment difficult [5]. According to Gondim Jr. et al. [6], diagnosis and treatment of root canals represent an additional challenge for the professional. Thus, a careful examination of preoperative radiographs and the use of computed tomography as an additional diagnostic tool will be important to detect anatomical variations [7, 8].

On the other hand, the development of NiTi endodontic files used in rotatory systems allows biomechanical preparation in a shorter period of time than the conventional manual files, despite the difficulties due to the complex variation in the root canal anatomy [1]. Moreover, the use of optical microscope in Endodontics has been increased over the course of years [9]. The optical microscope offers many benefits, such as great lighting and better visualization of the operative field [10]. The high magnification is needed to assist in locating calcified canals, detect root microfractures, identify isthmuses, assist in the posts removal in the field and interpret the complexities of the root canal system [11].

Therefore, the aim of this paper was to report a clinical case of endodontic treatment of a three-canal premolar using the ProTaper system and optical microscope.

## 2. Case Report

The patient, a 16-year-old girl, presented at the Dental Clinic of the Federal University of Amazonas to undergo endodontic treatment of tooth 14, reporting persistent pain after cold stimulation. Considerable destruction by caries was visible in the distal surface of the tooth. In the anamnesis, the patient reported no systemic or heart diseases and no use of medications or drugs.

**Figure 1:**
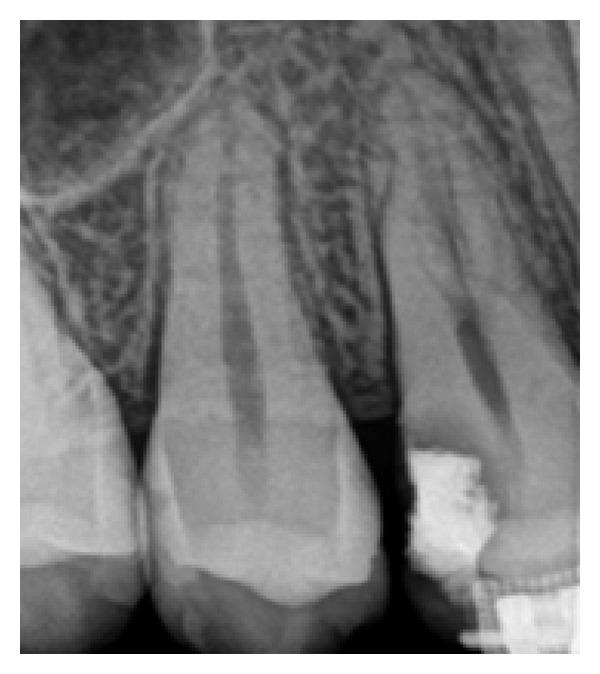
Initial periapical radiography. Radiopacity alteration in the middle third of the pulp chamber of tooth 14, suggesting trifurcation of the root canal.

**Figure 2:**
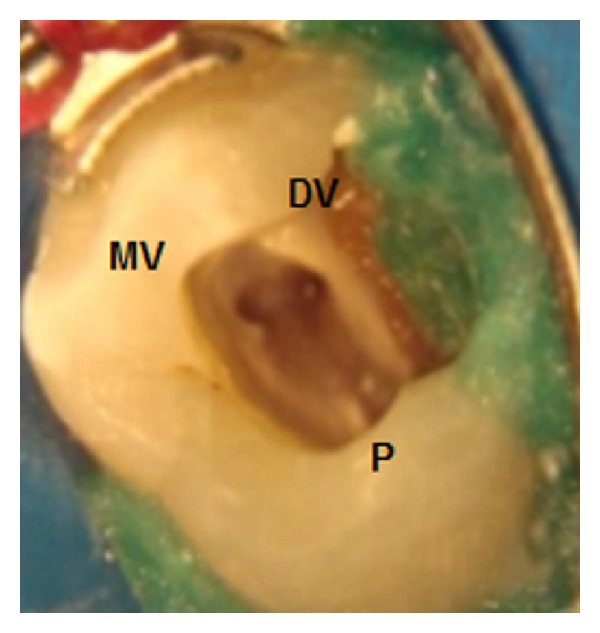
Root canal orifices located with operative optical microscope.

From the radiographic examination, an alteration in the radiopacity in the middle third of the pulp chamber of tooth 14 was observed, suggesting trifurcation of the root canal (Figure 1). The tooth was diagnosed as asymptomatic irreversible pulpitis and endodontic treatment was instituted. After local anesthesia (Xylestesin 2%, Cristália—Produtos Químicos Farmacêuticos Ltda., Itapira, SP, Brazil) and rubber dam isolation (Hygienic, Coltene/Whaledent AG, Alstatten, Switzerland) of the operative area, pulp chamber access was performed using a diamond spherical bur number 1013 (KG Sorensen, São Paulo, SP, Brazil). Next, the root canals orifices were located with number 10 K-file (Dentsply/Maillefer, Ballaigues, Switzerland), with the aid of an operative optical microscope (DF Vasconcelos, Valencia, RJ, Brazil) to facilitate visualization (Figure 2).

**Figure 3:**
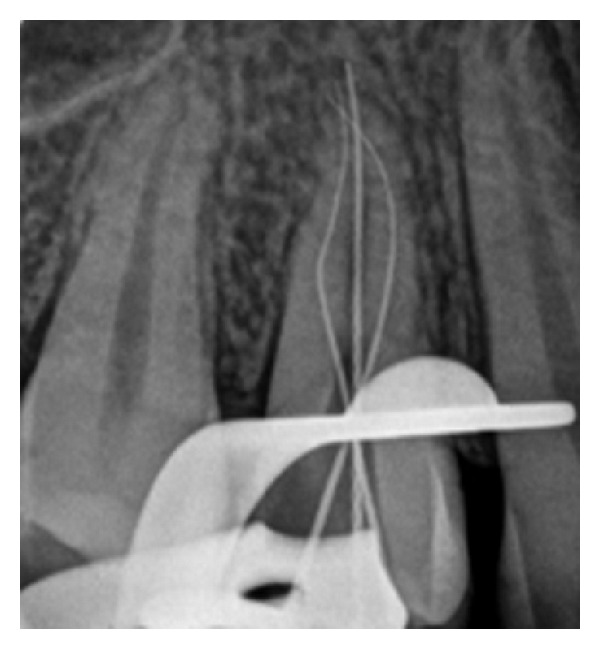
Odontometry confirming the presence of three root canals.

At each change of instrument, the canals were irrigated with 2.5 mL of 2.5% sodium hypochlorite solution (Biodinâmica, Ibiporã, PR, Brazil). During odontometry, the presence of three canals (Figure 3) was confirmed with the aid of an electronic apex locator (Joypex 5, Denjoy, China), two canals in the buccal root and one in the palatal root. The glide path was performed using PathFiles #13, #16, and #19 (Dentsply/Maillefer), cervical preparation with the ProTaper S1 and S2 instruments (Dentspy/Maillefer) and apical preparation with the ProTaper F1 and F2 instruments in the buccal canals and palatal canal up to the working length of 16 mm.

At the end of biomechanical preparation, 17% EDTA (Biodinâmica) was applied for 1 minute to remove smear layer and the final washing was performed with 2.5% sodium hypochlorite. Afterwards the root canals were dried with absorbent paper cones (Conetech, Manaus, AM, Brazil).

**Figure 4:**
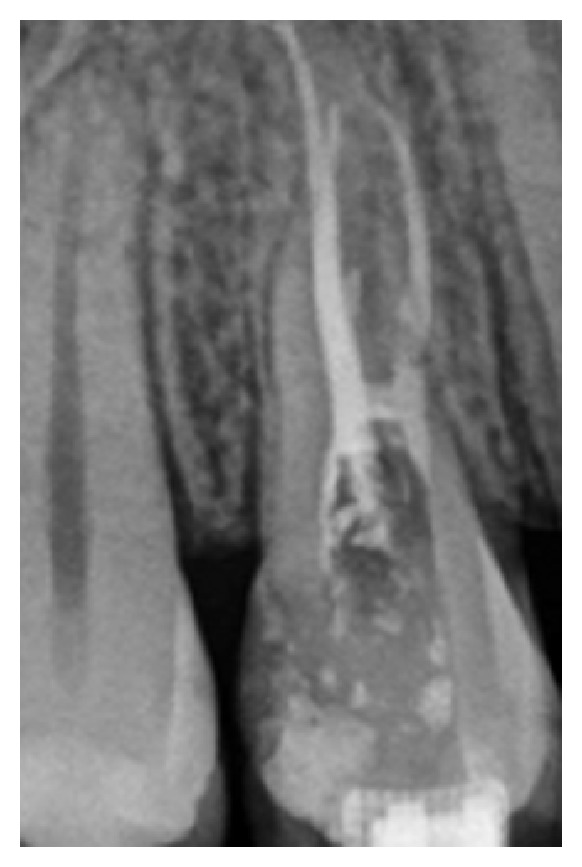
Final radiography after root canals filling.

The root canals were filled with Endofill sealer (Dentsply, Petrópolis, RJ, Brazil) and gutta-percha points F1 and F2 (Dentsply) by thermal compaction technique using McSpadden #50 (Dentspy/Maillefer). Next, final radiography was performed (Figure 4).

**Figure 5:**
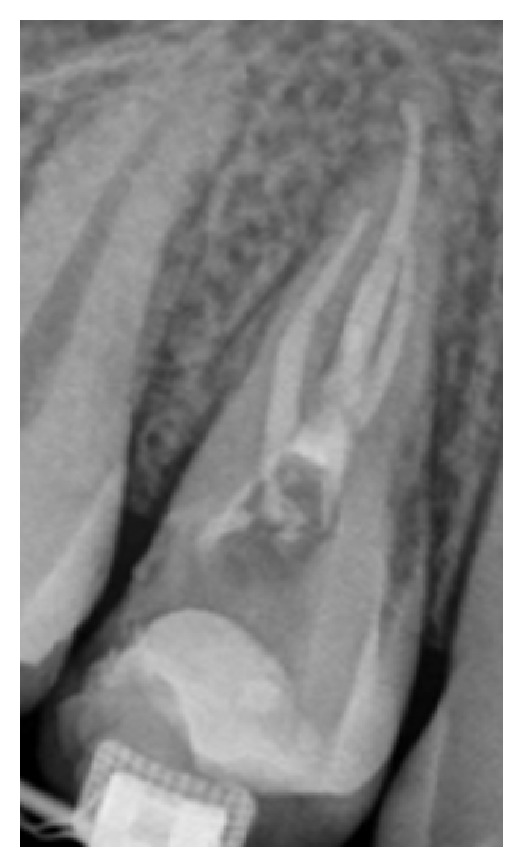
Periapical radiography of tooth 14 after 12 months, revealing no presence of failure in filling of root canals or periapical lesions.

The pulp chamber was cleaned to remove the excess of gutta-percha and sealer, and the tooth was temporarily restored with glass ionomer restorative cement (Ketac Molar, 3M ESPE, Sumaré, SP, Brazil). After 1 week, the tooth was definitely restored with composite resin (Z250, 3M ESPE, Sumaré, SP, Brazil). Follow-up over 12 months has shown a successful outcome from endodontic perspective. The tooth presented no painful symptomatology, and, radiographically, no signs of failure in root canal filling or periapical lesions were observed (Figure 5).

## 3. Discussion

An accurate diagnosis of the anatomy of the root canal system is a prerequisite for successful endodontic treatment [12]. Therefore, the radiographic signs that demonstrate the presence of anatomical variations must be considered an important condition when planning the tooth treatment [2].

Several studies have shown that the presence of three-rooted canals in maxillary first premolars is rare, ranging from 1.5% to 5% of the cases [2, 4, 5]. Although the frequency is low, the professional must be prepared to diagnose and plan a suitable treatment for this type of clinical event [2, 4, 5].

According to several case reports regarding the endodontic treatment of three-rooted maxillary premolars, it can be stated that, irrespective of the biomechanical preparation and filling technique used, the importance of early diagnosis of anatomical variations for satisfactory treatment outcome is imperative [13–16].

According to Vertucci [2], two radiographic signs indicate the presence of three-rooted maxillary first premolars. First, if it was radiographically detected that the middle third of the root has a mesiodistal distance equal to or greater than the cervical third, this might indicate that there are two buccal roots or two root canals in a single-wide buccal root [2]. The second radiographic sign is the rapid disappearance of the continuity of radiolucent image of the root canal [2]. In this clinical case, the sudden disappearance of the continuity of the radiolucent image related to the root canal, as observed in the initial radiograph (Figure 1), was suggestive of a third root canal.

To confirm the presence of the third canal in the maxillary premolar in this case, the operative optical microscope was used for improved clinical visualization. According to Karapinar-Kazandag et al. [11], the optical microscope has many advantages because it provides better illumination and visualization of the operative field with a magnification up to twenty times. Therefore, high magnification helps the identification of the anatomical details of the pulp chamber floor and orifices of the root canal entrance [11].

The PathFile instruments were used in this case for initial catheterization because they maintain the trajectory of the root canal and perform preenlargement for the shaping files. Within the limits of the study conducted by Berutti et al. [1], the NiTi rotary PathFiles seem to be appropriate tools for creating secure and easy catheterization before using the NiTi rotatory instruments to shape the canal. These files presented better maintenance of the original canal anatomy causing less modification of canal curvature [1].

Moreover, the ProTaper Universal system was chosen to perform instrumentation of the root canal system in this case due to one of its main characteristics: the rate of taper varies along the cutting flutes, which significantly enhances flexibility, allowing instrumentation of difficult areas with cutting efficiency and safety [17].

Therefore, this case report demonstrated that the use of technological tools such as optical microscopy and rotatory instrumentation systems is capable of assisting the endodontic treatment of teeth with complex internal anatomy such as premolars with three root canals.
